# The evolution of sampling in epidemiology: from classical probability models to AI-enhanced recruitment and active learning

**DOI:** 10.4178/epih.e2026019

**Published:** 2026-05-05

**Authors:** Sulaiman Abubakar Musa

**Affiliations:** Department of Planning, Research and Statistics, Federal Neuropsychiatric Hospital, Kano, Nigeria

**Keywords:** Sampling theory, Epidemiology, Artificial intelligence, Active learning, Algorithmic bias

## Abstract

Sampling is a foundational element of epidemiological research because it determines the validity, efficiency, and generalizability of population-based inferences. Classical probability and non-probability sampling methods have long underpinned public health study design by providing robust frameworks for unbiased estimation and causal inference. However, the rapid expansion of digital health data, electronic health records, and large-scale online cohorts has exposed important limitations in these largely static sampling frameworks. This narrative review traces the evolution of sampling in epidemiology from traditional probability-based designs to contemporary artificial intelligence-enhanced recruitment strategies, with particular emphasis on active learning and adaptive sampling. By synthesizing classical statistical theory with emerging machine-learning approaches and recent methodological advances, this review argues that the future of epidemiological sampling lies in hybrid frameworks that preserve statistical rigor while leveraging algorithm-driven adaptability. Methodological opportunities, ethical risks, and implications for low-income and middle-income countries are critically examined.

## GRAPHICAL ABSTRACT

[Fig f1-epih-48-e2026019]

## Key Message

• Epidemiological sampling is evolving from static, design-based probability frameworks toward dynamic, AI-enhanced systems that improve efficiency and responsiveness in large-scale studies.

• While classical sampling remains essential for inferential validity, integrating active learning and algorithmic recruitment enables researchers to prioritize high-information cases and navigate resource-constrained environments more effectively.

• The future of the field lies in hybrid frameworks that combine statistical rigor with adaptive machine-learning strategies, provided these are supported by robust ethical governance to mitigate algorithmic bias and address health inequities.

## INTRODUCTION

Sampling lies at the core of epidemiological research, shaping the validity, efficiency, and generalizability of population-based inferences. From early descriptive surveys to modern analytic and predictive models, representative sampling has remained a prerequisite for credible public health evidence [1,2]. Classical probability-based sampling designs, including simple random, stratified, cluster, and multistage sampling, have historically provided the statistical foundation for unbiased estimation and causal inference [3,4].

Alongside these approaches, non-probability and adaptive sampling methods have played an important role in studies of hidden or hard-to-reach populations, particularly in settings where complete sampling frames are unavailable. Respondent-driven sampling (RDS) extends snowball techniques by incorporating weighting schemes to improve population estimates among marginalized populations [5]. Methodological comparisons have further clarified the appropriate applications and limitations of convenience and purposive sampling in applied health research [6].

Despite their theoretical strengths, traditional sampling methods face increasing challenges in the contemporary epidemiological landscape. The proliferation of electronic health records, digital cohorts, mobile health platforms, and web-based surveys has fundamentally altered how populations are identified and accessed [7]. Declining response rates, rising recruitment costs, and persistent underrepresentation of marginalized groups have further strained classical sampling strategies [8]. At the same time, the scale and heterogeneity of modern health data have made static sampling designs increasingly inefficient for real-time surveillance and large observational studies [9].

Advances in artificial intelligence (AI) and machine learning offer new opportunities to address these challenges. AI-driven approaches can automate recruitment processes, prioritize high-information cases, and dynamically adapt sampling strategies as data accumulate [10,11]. Of particular relevance is active learning, a class of machine-learning techniques in which algorithms iteratively select the most informative observations for labeling or inclusion [12].

Conceptually, active learning aligns closely with classical sequential and adaptive sampling designs in statistics and epidemiology [13], suggesting a methodological bridge between traditional theory and modern computational approaches. Rather than positioning AI as a replacement for established sampling principles, this review argues that AI-enhanced recruitment strategies should be understood as extensions of classical epidemiological logic. However, these innovations raise important ethical and methodological concerns, including algorithmic bias, transparency, and the risk of exacerbating existing health inequities [14].

This review traces the evolution of sampling from classical probability models to AI-enhanced recruitment and active learning, with particular attention to hybrid frameworks applicable to low-income and middle-income countries (LMICs).

## MATERIALS AND METHODS

### Literature search strategy

This narrative review draws on literature from epidemiology, biostatistics, digital health, and machine learning. Searches were conducted in PubMed, Scopus, IEEE Xplore, and Google Scholar using combinations of keywords, including “sampling,” “epidemiology,” “probability sampling,” “non-probability sampling,” “adaptive sampling,” “artificial intelligence,” and “active learning.” Peer-reviewed articles published in English between 1960 and 2025 were considered, with priority given to seminal methodological texts and recent advances in digital and AI-enhanced approaches. Policy documents from major public health organizations were also included. Given the conceptual focus of the review, formal quality appraisal was not undertaken. Nigerian psychiatric and cardiovascular research was used as illustrative case material for implementation in low-resource settings, with quality-appraisal guidance drawn from the AXIS tool [15].

### Scope and limitations

Although this review used a structured search strategy, it was designed as a narrative synthesis rather than a formal systematic review or meta-analysis. Consequently, formal quality appraisal of the included literature was not performed. The scope of the review was limited to English-language publications, which may introduce geographic bias toward Anglophone research settings. Given the conceptual focus on the transition toward AI-enhanced frameworks, the review prioritizes literature that bridges statistical theory and computational practice. To support global relevance while retaining granular detail, regional data—particularly from Nigerian psychiatric and cardiovascular research—are used as illustrative examples for implementation in low-resource settings.

### Classical sampling methods in epidemiology (phase I)

#### Probability-based sampling

Probability sampling designs assign known, non-zero selection probabilities to all units in the target population, enabling unbiased estimation and valid inference. Common designs include simple random, stratified, cluster, and multistage sampling [3,4]. These approaches remain central to national surveys, cohort studies, and surveillance systems because of their strong theoretical guarantees of representativeness and inferential validity [1].

As established in foundational sampling theory [3], these designs provide strong theoretical guarantees of representativeness when properly implemented. A critical technical metric in this context is the design effect, a concept popularized by Kish [1]. In complex multistage or cluster designs, which are common in public health research, the design effect is the ratio of the actual variance to the variance expected under simple random sampling. Accounting for this effect is essential for maintaining precision and appropriately adjusting sample sizes when intracluster correlation is present [3].

#### Refining sampling in hidden populations

The evolution of phase I methods reached a sophisticated stage with the development of RDS. Unlike traditional snowball sampling, RDS uses a mathematical model based on Markov chains to reach a “steady state,” in which the final sample characteristics become independent of the initial “seeds” [5]. By applying weights that account for participants’ network sizes, also known as degree weighting, RDS can support population-level estimates even when a formal sampling frame is absent.

#### Non-probability and early adaptive approaches

Non-probability sampling methods, such as convenience, purposive, and snowball sampling, are frequently used when probability sampling is infeasible. Although these approaches lack design-based guarantees, they remain indispensable in exploratory research and studies of hard-to-reach populations [6]. Sequential sampling designs, which allow interim decisions about sample size and study continuation, represent early adaptive frameworks that anticipated later algorithm-driven approaches [13].

In Nigerian mental health epidemiology, RDS and similar chain-referral techniques have been valuable for studying stigmatized groups affected by psychiatric disorders. For example, community-based studies in southwestern Nigeria have examined perceptions of the causes of mental illness and treatment preferences, revealing high levels of stigma [16]. Nationwide surveys have further highlighted widespread beliefs about the manifestations of mental disorders, with many respondents attributing these conditions to possession by evil spirits [17]. These non-probability and adaptive approaches are particularly relevant in northern Nigeria, where they could facilitate recruitment for studies of community stigma in underserved populations.

### The digital shift in epidemiological sampling (phase II)

Digital technologies have reshaped sampling frames through electronic health records, administrative data linkage, web-based recruitment, and social media platforms. These developments have expanded the scale and efficiency of epidemiological sampling, but they have also introduced new forms of selection bias and coverage error [7]. In digital epidemiology, bias control often shifts from the design stage to post hoc adjustment and modeling, requiring careful methodological oversight [8].

In this phase, the focus shifts from design-based inference to model-based inference, in which representativeness is pursued through statistical adjustments after data collection [18]. To mitigate coverage errors in digital cohorts, researchers increasingly rely on sophisticated post hoc adjustment techniques. Propensity score weighting is frequently used to model an individual’s probability of inclusion on the basis of known covariates from a census-based reference population [18]. Similarly, the method of multilevel regression and poststratification allows researchers to reweight nonrepresentative digital samples to generate population-level inferences [18]. As epidemiology increasingly uses administrative data linkage, the technical challenge shifts toward maintaining data quality across disparate systems and managing missing identifiers through robust preprocessing pipelines [7].

In Nigeria, digital approaches are emerging for maternal mental health surveillance amid stigma and barriers to care. For example, surveys of perinatal adolescents in Ibadan have documented high mobile phone ownership (89.6%) and strong interest in using short message service (SMS)/text messages or applications (apps) for mental health information, with interest exceeding 93% [19]. Recent studies of postpartum women have examined mobile apps, teletherapy, AI-driven chatbots, and SMS-based interventions for postpartum depression (PPD), highlighting their effectiveness in rural settings, including structured programs that reduced PPD symptoms by 15% in northern rural communities [20]. Such digital recruitment and data-collection strategies shift bias control toward post hoc modeling but offer scalability for psychiatric epidemiology in facilities such as Federal Neuropsychiatric Hospital Dawanau, where incomplete registries limit traditional sampling frames. The integration of large-scale digital data has also intensified interest in scalable analytic tools, although concerns remain about representativeness and ethical governance in LMIC settings where digital divides persist [21].

### Artificial intelligence -enhanced recruitment and active learning (phase III)

#### Active learning frameworks

To ensure methodological clarity in this phase, 3 closely related concepts should be distinguished. Adaptive sampling refers to classical statistical designs in which selection depends on previously observed values [13]. Active learning, by contrast, is a specific subfield of machine learning in which an algorithm proactively queries or selects the most informative data points for labeling to maximize model performance while using the smallest feasible training set [12]. This differs from AI-enhanced recruitment, which focuses on the operational efficiency of identifying and enrolling participants through predictive modeling [11,22].

Active learning refers to machine-learning strategies that iteratively select observations expected to yield the greatest informational gain. Common strategies include uncertainty sampling, query-by-committee, and information-density methods [12]. Comprehensive surveys have demonstrated how deep and stream-based active learning approaches can scale to high-dimensional and continuously generated data environments characteristic of modern health systems [23,24]. In epidemiological contexts, active learning parallels targeted case finding and adaptive surveillance, enabling efficient allocation of limited resources. Recent hybrid integrations further highlight opportunities to combine AI with mechanistic models [25].

#### Recruitment efficiency, imbalance, and fairness

AI-enhanced recruitment models have been developed to predict enrollment likelihood, dropout risk, and outcome relevance, thereby enabling targeted oversampling of underrepresented populations [11,22]. However, algorithmic decision-making introduces risks of bias amplification when training data reflect existing inequities. Extensive reviews of bias and fairness in machine learning highlight the need for systematic bias identification and mitigation throughout the model lifecycle [14].

Emerging AI apps in Nigerian healthcare demonstrate practical potential for improving recruitment efficiency in resource-constrained settings. The SPEC-AI Nigeria trial used AI-guided screening with digital stethoscopes and electrocardiography and doubled the detection of peripartum cardiomyopathy in obstetric populations, improving case finding with a low number needed to screen [26]. In mental health, Nigerian psychiatrists and trainees have expressed optimism that AI could help address workforce shortages; research indicates high readiness among these professionals to integrate AI-driven diagnostic support and screening tools [27]. Concerns about transparency and interpretability remain salient; however, evidence from healthcare AI research indicates that interpretable models can achieve high predictive performance while supporting ethical and accountable decision-making [28,29].

### Methodological comparison of sampling phases

The evolution of sampling in epidemiology can be conceptualized as a transition across 3 interrelated paradigms: classical probability-based sampling, digitally mediated sampling, and AI-enhanced adaptive sampling. Classical approaches emphasize design-based inference and randomization. Digital sampling improves scalability but often relies on model-based bias correction. AI-enhanced sampling introduces dynamic, information-driven selection embedded within predictive systems, and emerging hybrid models offer enhanced forecasting and adaptability [22]. Hybrid frameworks that integrate probabilistic foundations with adaptive algorithms offer the most robust pathway forward by balancing efficiency with inferential validity.

Table 1 presents a conceptual synthesis developed by the author based on multiple sources; it is not adapted from a prior publication.

### Ethical considerations for artificial intelligence-enhanced sampling

AI-enhanced sampling raises critical ethical challenges related to fairness, accountability, transparency, and equity. International policy frameworks emphasize that AI systems in health should augment, not undermine, public trust and social justice. Global guidance from the World Health Organization highlights the importance of governance structures, human oversight, and equity-centered design in AI-assisted health research [30,31]. Particular attention is needed in LMICs, where AI risks exacerbating disparities if training data reflect existing inequities [32].

## IMPLICATIONS FOR LOW-INCOME AND MIDDLE-INCOME COUNTRIES

In LMICs, epidemiological research is often constrained by limited resources, incomplete registries, and fragmented health information systems. While these constraints challenge classical sampling, they also highlight the potential value of adaptive and AI-enhanced approaches that prioritize efficiency. However, without careful governance, such approaches risk reinforcing digital exclusion and structural inequities, including through biased algorithms [30]. Investment in infrastructure, capacity building, and ethical oversight is therefore essential.

In northern Nigeria, where psychiatric services face severe shortages and stigma persists, facilities could pilot hybrid approaches—combining classical RDS for community outreach with digital or mobile tools for follow-up and AI for prioritizing informative cases in surveillance or research. This would address incomplete registries and resource constraints while advancing equitable mental health evidence generation.

## CONCLUSION

The evolution of sampling in epidemiology reflects a shift from static, design-based frameworks toward adaptive, data-driven strategies. Classical probability sampling remains indispensable for valid inference, while AI-enhanced recruitment—particularly through active learning—offers powerful tools for improving efficiency and responsiveness. The future of epidemiological sampling lies in hybrid frameworks that integrate statistical rigor, algorithmic adaptability, interpretability, and ethical governance, with careful consideration for equitable application in diverse global contexts.

### Ethics statement

No ethical approval or informed consent were not required since this study was based on published articles only, and did not involve any human subject data.

## Figures and Tables

**Figure f1-epih-48-e2026019:**
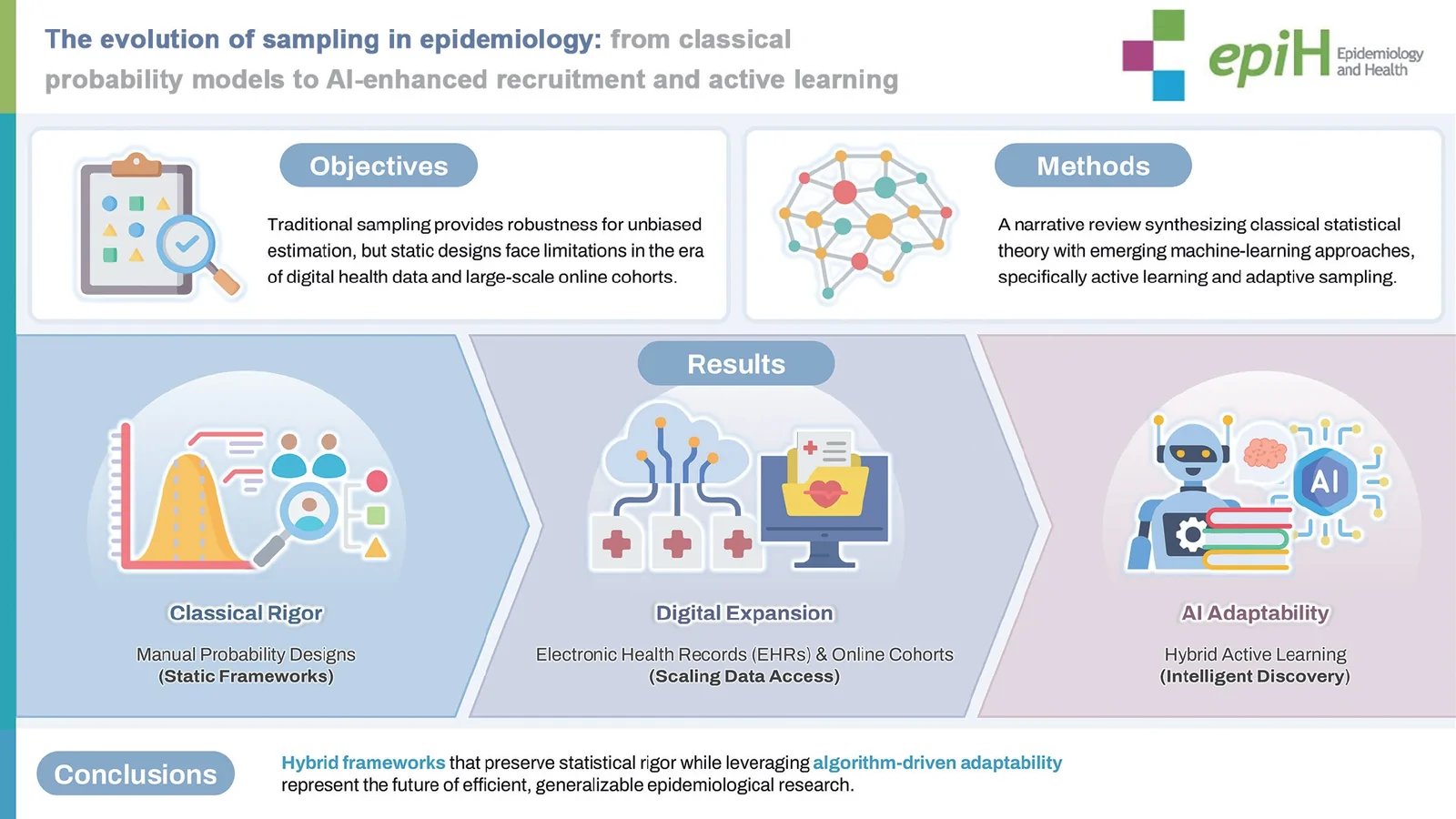


**Table 1.** The 3 phases of epidemiological sampling and the recommended hybrid future

Aspects	Phase I: Classical probability-based sampling	Phase II: Digitally mediated sampling	Phase III: AI-enhanced adaptive sampling	Hybrid frameworks (recommended future)
Core emphasis	Design-based inference and randomization	Scalability and large-scale data access	Dynamic, information-driven selection	Integration of probabilistic foundations with adaptive algorithms
Key methods	Simple random, stratified, cluster, and multistage sampling; non-probability methods, including RDS for hidden populations	Electronic health record linkage, web-based recruitment, social media, and mobile platforms	Active learning, including uncertainty sampling and query-by-committee; predictive recruitment models; adaptive surveillance	Probabilistic and AI-based methods, including active learning with mechanistic models [25]
Inference approach	Design-based inference using known selection probabilities	Often model-based, using post hoc adjustments	Algorithmic and predictive, focused on maximizing information gain	Combined design- and model-based inference that balances rigor and adaptability
Strengths	Strong theoretical guarantees of representativeness; valid causal inference	Expanded scale, efficiency, and real-time access	Prioritization of high-information cases; resource efficiency; dynamic adaptation	Enhanced forecasting, efficiency, and inferential validity; ability to bridge static and dynamic needs
Limitations	Static designs; inefficiency for large or heterogeneous data; high costs; declining response rates; difficulty studying hidden or hard-to-reach groups	Selection and coverage bias; reliance on post hoc modeling; digital divides, especially in LMICs	Risk of algorithmic bias amplification; black-box concerns; need for careful bias mitigation	Requires careful integration to avoid compounding bias
Bias handling	A priori, at the design stage through randomization and stratification	A posteriori, through modeling adjustments and weighting	Across the model lifecycle, including fairness-aware training	Systematic bias management across design, modeling, and algorithmic stages
Relevance to LMICs	Feasible when registries exist but constrained by incomplete frames and limited resources	Scalable but may exacerbate digital exclusion	High potential for efficiency in resource-constrained settings, including targeted case finding	Most promising when designed to address resource constraints while preserving equity, including pilots at facilities such as Dawanau
Examples from literature	National surveys and cohort studies [1,3]	Digital cohorts and web surveys [7]	Active learning in surveillance and targeted recruitment [11,12]	AI-mechanistic hybrid models for forecasting [25]
Suitability	Best suited for valid, unbiased population inference when sampling frames exist	Useful for rapid surveillance and hypothesis generation	Useful for real-time, resource-limited scenarios	Optimal pathway for combining efficiency, rigor, and equity

AI, artificial intelligence; RDS, respondent-driven sampling; LMICs, low-income and middle-income countries.

## Data Availability

No primary data were generated or analyzed for this study. All information is derived from publicly available sources cited in the manuscript.
